# Skin Grafting

**Published:** 1878-10

**Authors:** 


Skin-Grafting.—M. Maurel (*Le Proges Medical*, 29 juin, 1878) has performed skin-grafting on people of the white, yellow and black races. The operation succeeded in all, but on account of the thicknness in the derma, it is rather difficult to perform in the negro. Hetero-grafting (grafts taken from one race to another) has given interesting results. Their efficiency in the cure of wounds and ulcers is undoubted. The pigment disappears from the graft taken from a black and implanted upon a white person; This does not hold good for Negroes and Hindoos. When both subjects are strongly pigmented, the graft remains colored, and even by a sort of contact action, pigment is reproduced in the cicatrix in a very narrow zone surrounding the graft, outside of which the cicatrix is white, as is always the case in Negroes after large losses of substance. —*St. Louis Record.*

